# Recherche sur les maladies infectieuses dans le Service de santé des armées: cent ans après Alphonse Laveran

**DOI:** 10.48327/mtsi.v3i1.2023.333

**Published:** 2023-03-14

**Authors:** Jean-Nicolas Tournier

**Affiliations:** 1 Département Microbiologie et maladies infectieuses, Institut de recherche biomédicale des armées (IRBA), 1 place du Général Valérie André, 91220 Brétigny-sur-Orge, France; 2 École du Val-de-Grâce, 1 place Alphonse Laveran, 75005 Paris, France; * Actes du Colloque – Centenaire de la mort d'Alphonse Laveran. 24 novembre 2022, Paris / Proceedings of the Conference – Centenary of the death of Alphonse Laveran. 24 November 2022, Paris

**Keywords:** Alphonse Laveran, Risque infectieux, Histoire de la médecine, SSA, Service de santé des armées, France, Alphonse Laveran, Risk of infection, History of medicine, SSA, French Defence Health Service, France

## Abstract

Les armées ont toujours été particulièrement exposées au risque infectieux, ce qu'Alphonse Laveran analysa dès 1875 dans son *Traité des maladies et épidémies des armées.* De nos jours, le risque infectieux est toujours aussi présent, c'est pourquoi le Service de santé des armées (SSA) emploie dans ce domaine des moyens de recherche modernes structurés autour de l'Institut de recherche biomédicale des armées (IRBA) appuyé par les Hôpitaux d'instruction des armées (HIA), le Centre d’épidémiologie et de santé publique des armées (CESPA), et l’École du Val-de-Grâce.

Ces moyens répondent aux besoins actuels de recherche en maladies infectieuses et tropicales et se préparent à répondre aux futures émergences.

Récemment, la recherche du SSA a pu s'illustrer dans plusieurs épidémies et émergences qui ont touché les forces armées et la population nationale.

## Abstract (en)

The army has always been particularly exposed to the risk of infection, which Alphonse Laveran already analyzed in 1875 in his *Traité des maladies et épidémies des armées.* Nowadays, the risk of infection is still present, which is why the Armed Forces Health Service (SSA) employs modern research resources in this area structured around the Armed Forces Biomedical Research Institute (IRBA) supported by the Military Training Hospitals (HIA), the Armed Forces Epidemiology and Public Health Center (CESPA), and the Val-de-Grâce School.

These resources meet current research needs in infectious and tropical diseases and are preparing to respond to future emergences.

Recently, the SSA research has stood out in several epidemics and emergences that have affected the French Armed Forces and the national population.

## Introduction

Les armées ont toujours été particulièrement exposées au risque infectieux. Il suffit de flâner dans les couloirs du cloître de l’École du Val-de-Grâce devant les plaques commémoratives de marbre noir pour se convaincre des hécatombes provoquées par les maladies infectieuses dans les rangs des soignants. Sur les champs de bataille, celles-ci fauchaient massivement et indifféremment soignants et soignés. Alphonse Laveran fut le premier à le remarquer clairement en posant la question dans son *Traité des maladies et épidémies des armées* publié en 1875: « Pourquoi des hommes choisis avec soin, bien vêtus, régulièrement nourris, attentivement surveillés, payent à la mort un tribut plus lourd que les autres citoyens [7]? » Avant la découverte de l'hématozoaire du paludisme qui le fera passer à la postérité, Alphonse Laveran démontrait ses qualités scientifiques d’épidémiologiste de la communauté militaire dans cet ouvrage qui reste une référence de cette époque. Son traité de 1875 décrivait clairement, chiffres à l'appui, ce paradoxe de la surmortalité des jeunes militaires par rapport à la population civile. Il observait dans les années 1860 à 1875 un taux de mortalité moyen au sein des armées d'environ 16 pour 1000, soit 1,6%, principalement lié à des maladies infectieuses. Il faut se rappeler qu’à l’époque la tuberculose (dont le germe ne sera identifié par Robert Koch qu'en 1882) était la première cause de mortalité en métropole avec un taux de 5,33 pour 1 000 dans les forces armées de métropole. Alphonse Laveran remarquait par ailleurs que pour les armées stationnées en Algérie, la première cause en revenait aux fièvres palustres devant la phtisie et les fièvres typhoïdes. Cette forte prévalence de la malaria en Algérie justifiera quelques années plus tard l'intérêt d'Alphonse Laveran pour ses études sur l'origine de cette maladie.

La réponse à la question posée en exergue par Alphonse Laveran en 1 875 est désormais connue: le brassage, le rassemblement de populations, la promiscuité, les déplacements et les conditions rustiques, qui sont les bases de la vie en collectivité militaire, sont autant de facteurs favorisant la diffusion des maladies infectieuses. Ces facteurs sont très connus par les modélisateurs modernes des épidémies. La vie militaire est *per se* une cause amplificatrice des maladies infectieuses.

Cent ans plus tard, qu'en est-il? Les maladies infectieuses ont-elles disparu du champ des préoccupations de la médecine militaire? La réponse est clairement non, à la simple observation des données de la surveillance épidémiologique dans les armées en 2022. L'infectiologie reste toujours une des disciplines médicales régaliennes du Service de santé des armées (SSA). Ces maladies infectieuses sont mieux contrôlées par la vingtaine de vaccins inscrits au calendrier vaccinal des militaires, mais elles n'ont majoritairement pas été éradiquées en dehors de la variole.

Quel type de recherche en infectiologie est donc mené par le SSA? Nous répondrons à cette question en présentant tout d'abord les moyens et les structures au sein desquelles est effectuée cette recherche, avant de nous interroger sur les besoins de recherche en 2022, et d'illustrer par quelques exemples les actions de recherche récentes les plus saillantes.

## Moyens Et Structures De La Recherche En Infectiologie Dans Le Service De Santé des Armées

Quelles que soient leurs typologies, les recherches biomédicales de défense sont toutes menées au sein d'une institution unique: l'Institut de recherche biomédicale des armées (IRBA). Au sein de l'IRBA, trois grands types d'activités sont menées: recherche, enseignement et expertise appliquée aux armées. La recherche menée a ainsi comme fonction secondaire d'irriguer et de tirer vers l'excellence l'enseignement donné et l'expertise rendue. Par ailleurs, les recherches sont menées en réseau associant étroitement cliniciens, épidémiologistes et biologistes.

Pour le domaine de l'infectiologie, les recherches sont structurées au sein de deux pôles géographiques: un réseau situé en région Île-de-France orienté sur les maladies hautement pathogènes, et un réseau situé en région Provence-Alpes-Côte d'Azur (PACA) orienté sur les maladies tropicales.

Sur le pôle dédié aux maladies hautement pathogènes, la recherche est organisée autour des infrastructures de laboratoires confinés de l'IRBA et des deux Centres nationaux de référence (CNR) Charbon et Orthopoxvirus. À ces infrastructures sont associés les Hôpitaux d'instruction des armées (HIA) Bégin et Percy, avec notamment le service de maladies infectieuses et tropicales de l'HIA Bégin doté de deux chambres à dépression permettant d'accueillir des patients hautement contaminés, et les services de biologie clinique. Enfin, l’École du Val-de-Grâce complète le dispositif avec les enseignements du Master Gestion des risques NRBC (nucléaires, radiologiques, biologiques et chimiques) et la semaine NRBC à destination des praticiens et paramédicaux du service.

Sur le pôle de recherche localisé en région PACA, deux unités de recherche de l'IRBA dédiées au paludisme et aux arbovirus, porteuses des CNR Paludisme zone sud et Arbovirus, sont présentes au sein de l'Institut hospitalo-universitaire (IHU) Méditerranée Infection. Les HIA Laveran et Sainte-Anne complètent le dispositif clinique à travers leurs services de maladies infectieuses et tropicales et de biologie clinique. Le Centre d’épidémiologie et de santé publique des armées (CESPA) basé à Marseille, dont les praticiens participent à des Unités mixtes de recherche à l'IHU, complète le dispositif en épidémiologie et santé publique à travers ses compétences sur le territoire national ainsi qu'avec les forces prépositionnée et les forces en opérations extérieures.

Le SSA dispose ainsi d'un réseau cohérent de recherche sur les maladies infectieuses inséré plus largement dans les grands réseaux et instituts nationaux de recherche comme l'Institut Pasteur, l'Institut national de la santé et de la recherche médicale (INSERM), le Commissariat à l’énergie atomique et aux énergies alternatives (CEA) et l'université Paris-Saclay pour la plateforme Île-de-France, l'Institut pour la recherche et le développement (IRD), l'université Aix-Marseille et l'INSERM pour la plateforme PACA.

## Besoins De Recherche En 2022

Comme le cherchait intuitivement Alphonse Laveran dans son *Traité des maladies et épidémies des armées*, les besoins de recherche sont orientés par les déterminants épidémiologiques et les risques auxquels sont exposés les militaires. Depuis 2018, dans le SSA, les objets des actions de recherche sont encadrés par le plan d'orientation de la recherche et de l'innovation du SSA. En termes d'infectiologie, les actions portent d'une part sur les maladies infectieuses tropicales dont le paludisme reste d'actualité en 2022. Ces actions sont sous-tendues par le CNR Paludisme dont l'objet est la surveillance de la sensibilité à la chimioprophylaxie. En termes de maladies tropicales, le second axe concerne les arbovirus, dont les recherches sont aussi sous-tendues par les activités du CNR Arbovirus. Les actions de recherche portent d'autre part sur les agents hautement pathogènes dont les actions sont aussi supportées par les CNR Charbon et Orthopoxvirus.

Par ailleurs, depuis le début du xxi^e^ siècle, une tendance épidémiologique lourde a été l'accélération des émergences virales. Ainsi le prophétisait Charles Nicolle, un autre prix Nobel de physiologie ou de médecine, dans ses leçons au Collège de France: « Il y aura donc des maladies nouvelles. C'est un fait fatal. Un autre fait, aussi fatal, est que nous ne saurons jamais les dépister dès leur origine [10]. » Cette phrase ciselée, dépouillée, aux allures si modernes, presque prophétiques, a été écrite il y a un peu moins d'un siècle, à une époque où le dénombrement et l'identification de toutes les maladies infectieuses étaient loin d’être achevés, notamment pour les maladies virales pour lesquelles nous ne disposions pas de moyen de culture. Avec l’émergence de la COVID-19 en 2020, la prise en compte de la problématique des émergences est devenue une évidence pour tous. Les facteurs anthropogéniques qui favorisent celles-ci ne vont que s'accélérer avec l'entrée de l'humanité dans l’ère de l'Anthropocène. Il est donc certain que les maladies émergentes vont constituer une aire de recherche en croissance dans les années futures, notamment pour le SSA qui est sollicité pour participer à la résilience de la nation comme il l'a été en 2020 pendant la pandémie de COVID-19.

## Actions De Recherche Au Profit Des Armées: Quelques Exemples Saillants Récents

Faute de place, nous ne pourrons être exhaustif dans cet article à propos de la recherche au sein du SSA. Aussi avons-nous choisi de le faire à travers trois exemples récents à propos de trois évènements émergents.

### Émergence d’*Anopheles stephensi* à Djibouti en 2019

La France disposant d'accords de défense avec la République de Djibouti, les armées françaises stationnent des forces sur plusieurs emprises réparties sur la ville de Djibouti. La situation épidémiologique du paludisme à Djibouti a fortement évolué sur les dix dernières années. Avant 2013, le paludisme était hypo-endémique dans le pays, donnant un espoir d’éradication, avec de faibles niveaux de transmission en périphérie urbaine et dans les zones rurales de décembre à mai. La plupart des cas étaient causés par une infection à *Plasmodium falciparum.* Avant 2013, le moustique *Anopheles arabiensis* était le principal vecteur du paludisme. À partir de 2014, l'incidence du paludisme est progressivement remontée. En 2018, son incidence est passée à 25 319 cas confirmés (64% causés par *P. falciparum* et 36% par *P. vivax*) et probablement plus de 100 000 cas suspects. Devant l'augmentation des cas, la Direction centrale du SSA a diligenté une enquête épidémiologique associant des épidémiologistes du CESPA et des chercheurs de l'IRBA. Réalisée avec capture et identification de larves et d'adultes, cette enquête a permis d'identifier *A. stephensi* sur l'ensemble des sites prélevés [4]. Ce vecteur originaire d'Asie a plusieurs inconvénients dont celui de pouvoir proliférer en zone urbaine, et d’être résistant à la majorité des insecticides utilisés pour la lutte antivectorielle. Il est donc susceptible de changer dans une large mesure le faciès épidémiologique du paludisme. Après son identification à Djibouti, *A. stephensi* a été signalé en Éthiopie en 2016, au Soudan et en Somalie en 2019, et au Nigéria en 2020. Une étude prédictive a déterminé que plus de 126 millions de personnes supplémentaires dans les grandes agglomérations seraient à l'avenir exposées au risque de paludisme transmis par *A. stephensi* [12]. À l'automne 2022, de nombreux auteurs se sont interrogés sur les conséquences de cette émergence [11], dont l'entrée sur le continent africain avait été identifiée dès 2019 par les chercheurs du SSA.

### Émergence de la COVID-19 à bord d'un porte-avions en 2020

Le 21 janvier 2020, le groupe aéronaval (GAN) constitué du porte-avions Charles de Gaulle (PAN CDG) et de son escadre a appareillé de Toulon pour la mission opérationnelle « Foch 2020 ». À la date du départ du GAN, aucun cas de COVID-19 n'avait été déclaré en France. Le PAN CDG a ensuite effectué une escale à Chypre en janvier et une autre à Brest en mars 2020. Malgré certaines mesures prises à bord pour éviter l'introduction du virus, une alerte épidémique est lancée le 7 avril 2020. Une première équipe d'investigation mêlant épidémiologistes et chercheurs de l'IRBA a été déployée le 8 avril 2020 sur le PAN CDG, au large du Golfe de Gascogne [8]. Des prélèvements nasopharyngés ont été réalisés à bord par l’équipe d'investigation, permettant de confirmer biologiquement la présence du virus SARS-CoV-2 par l'IRBA chez 66 marins malades. Au final plus de 1 000 marins seront diagnostiqués positifs à la COVID-19. L'enquête épidémiologique qui a suivi sous la responsabilité du CESPA a porté sur une population de 1568 marins, constituant la population d’étude. Elle a permis de confirmer une introduction du virus entre Chypre et Brest [3]. Ce sont toutefois les analyses phylogénétiques réalisées à l'IRBA qui ont pu clairement affirmer l'existence de plusieurs clusters de contamination liés à plusieurs variants. Les variants ont été apparentés à des souches précédemment isolées dans les régions Bretagne, Île-de-France et Hauts-de-France. Ces résultats étaient donc compatibles avec une introduction du virus par plusieurs marins infectés et contagieux.

Cette épidémie qui avait eu un fort retentissement médiatique n'a en fait rien d'exceptionnel au regard de la promiscuité quotidienne des équipages en milieu maritime. De tels environnements catalyseurs de la transmission virale ont été décrits pour la COVID-19 en secteur civil pour le navire de croisière Diamond Princess [9] et en secteur militaire sur le porte-avions nucléaire américain USS Theodore Roosevelt [6].

Pour les missions opérationnelles de 2021 et 2022, l'IRBA a été à nouveau sollicité pour aider la Marine nationale à mettre en place à bord du PAN CDG un système de surveillance des eaux noires à bord, sur le modèle du réseau de surveillance OBEPINE au niveau national [1].

### Émergence du virus Monkeypox en population générale en 2022

Début mai 2022, alors que le monde de la virologie était encore focalisé sur la course haletante du SARS-CoV-2 autour de la planète, un nouveau venu a fait sa brusque apparition en Europe chez l'homme: le virus Monkeypox (MPXV), finalement rebaptisé virus Mpox par l'Organisation mondiale de la santé le 28 novembre 2022 pour éviter la stigmatisation des communautés dans lesquelles il a émergé. Dans ce contexte, le CNR Orthopoxvirus de l'IRBA a été sollicité pour effectuer le diagnostic du premier cas français. Sur ce patient, la souche a été isolée, son génome a été rapidement caractérisé, et sa sensibilité au tecovirimat validée permettant d'ouvrir la voie à son utilisation en santé humaine [5].

Si cette épidémie semble sous contrôle clinique actuellement, il n'est pas évident que des récurrences de ce virus ou sa diffusion dans des populations naïves soient exclues. Cette émergence brutale pose la question de manière plus large du développement de solutions vaccinales dirigées contre le MPXV et aussi son proche cousin, le virus de la variole.

## Perspectives

Les médecins militaires de 2022, et ceux des générations futures, comme leur prédécesseur illustre, le professeur Alphonse Laveran, auront à affronter le péril infectieux. C'est une évidence. Seuls les moyens et les outils pour aborder le problème ont progressé en un siècle. En 2022, la recherche en infectiologie est plus structurée qu’à l’époque d'Alphonse Laveran avec un Institut de recherche, un réseau d'HIA, le CESPA, l’École du Val-de-Grâce pour ne citer que les acteurs au sein du SSA. En dehors du Service de santé des armées, cette recherche est connectée aux grands instituts nationaux du domaine que sont l'Institut Pasteur, le CEA, l'INSERM et l'IRD.

Les générations futures de praticiens militaires et de paramédicaux disposeront donc d'outils de recherche mieux structurés pour répondre aux problématiques des futures émergences. Toutefois, la relation singulière du médecin face à une épidémie restera fondamentalement la même, comme nous l'a rappelé Albert Camus: « Le fléau n'est pas à la mesure de l'homme, on se dit donc que le fléau est irréel, c'est un mauvais rêve qui va passer. Mais il ne passe pas toujours et, de mauvais rêve en mauvais rêve, ce sont les hommes qui passent, et les humanistes en premier lieu, parce qu'ils n'ont pas pris leurs précautions [2]. »

## Liens Intérêts

L'auteur ne déclare aucun lien d'intérêt.
